# Phoxim induces neurotoxicity and intestinal damage in *Caenorhabditis elegans*

**DOI:** 10.3389/fphar.2025.1657527

**Published:** 2025-12-09

**Authors:** Mengyuan Zhang, Xin Zhao, Hua Bai, Qi Wang, Wei Zou

**Affiliations:** Department of Public Health Kunming Medical University, Kunming, China

**Keywords:** phoxim, *Caenorhabditis elegans*, autophagy, neurotoxicity, intestinal damage

## Abstract

**Introduction:**

Phoxim (chemical name O-α-cyanophenylamino-O, O-diethyl phosphorothioate, molecular formula C_12_H_15_N_2_O_3_PS) is classified as a high-efficiency, low-toxicity organophosphorus insecticide. Its primary mechanism of action involves inhibition of cholinesterase activity in insects, which disrupts nerve conduction, ultimately leading to paralysis and death.

**Methods:**

The effects of phoxim exposure (0.5, 1, and 2.5 µg/mL) on survival, neurological function, and intestinal integrity in *Caenorhabditis elegans (C.elegans)* were investigated.

**Results:**

Phoxim at all concentrations significantly increased the mortality rate of *C. elegans.* Fluorescence microscopy revealed that 2.5 µg/mL phoxim reduced dopaminergic neural processes in the BZ555 transgenic strain of *C. elegans* from 4 to 2, and 0.5 and 1 µg/mL phoxim accelerated amyloid beta (Aβ)-induced paralysis in the CL4176 strain, with complete paralysis observed at 32 and 36 h, respectively. FD&C Blue #1 staining demonstrated intestinal damage in 46.7% and 68.3% of *C. elegans* exposed to phoxim at 1 and 2.5 µg/mL, respectively. Exposure to 1 µg/mL phoxim decreased enterocyte numbers and reduced autophagic vesicles in the *lgg-1*::GFP strain of *C. elegans* from 1.8 to 1.3. qPCR analysis revealed downregulation of autophagy-related genes (*vps-34, atg-13*, and *unc-51*) by 0.53-, 0.43-, and 0.36-fold of the control levels, respectively. RNAi targeting the *eat-2* gene further confirmed the impact of phoxim on cell survival through the autophagy pathway.

**Discussion:**

Our results indicate that phoxim exposure reduces dopaminergic neuron integrity, accelerates Aβ-induced paralysis, and damages intestinal cells through inhibition of autophagy in *C. elegans*.

## 1 Introduction

Phoxim (chemical name O-α-cyanophenylamino-O, O-diethyl phosphorothioate; molecular formula C_12_H_15_N_2_O_3_PS) is classified as a high-efficiency, low-toxicity organophosphorus insecticide. Its primary mechanism of action involves the inhibition of cholinesterase activity in insects, which disrupts nerve conduction, ultimately leading to paralysis and death. In addition, phoxim inhibits host cholinesterase activity, enhances gastrointestinal peristalsis, and accelerates parasite expulsion from host organisms (Li and Liu, 2019; Wang et al., 2020). The versatility of phoxim has been demonstrated by its broad insecticidal spectrum and the ease of application. It effectively controls various lepidopteran larvae and other agricultural pests (Lin et al., 2011; Zhang et al., 2023; Yu and Li, 2015). In the aquaculture industry of China, phoxim is widely used as a treatment for parasitic infections, including species of *Gyrodactylus*, *Dactylogyrus*, and *Trichodina*, and it plays a crucial role in pond sanitization and disease management (Tang et al., 2015; Zhang et al., 2015; Gao et al., 2014).

Despite the beneficial applications in pest control, phoxim poses significant environmental challenges. When applied in agricultural fields, it can enter surface water systems via runoff, resulting in aquatic contamination. This contamination has become a serious constraint on the further development of the aquaculture industry in China and aquatic product exports (He et al., 2021). The ecological impact of phoxim extends to various aquatic species, with documented toxic effects on juvenile rainbow trout (Tang et al., 2015), goldfish (Shang and Zhang, 2015), black-tailed near-red bleaks (Xu et al., 2013), and large-bodied *Paralichthys olivaceus* (Guan et al., 2020). At the molecular level, phoxim acts as an inhibitor of the CYP3A enzyme activity, mRNA expression, and protein production in the liver of crucian carp.

*C*. *elegans* has emerged as a valuable model organism in toxicological research because of its numerous advantages, including a short life cycle, transparent body, fully sequenced genome, and a well-characterized nervous system comprising exactly 302 neurons (Okoro et al., 2021; Yu et al., 2021). The simple yet complete biological system of *C. elegans* makes it particularly suitable for high-throughput screening and mechanistic studies on environmental toxicants. Furthermore, approximately 60%–80% of human genes have homologs in *C. elegans*, enhancing the translational relevance of the findings from this model (Kaletta and Hengartner, 2006; Leung et al., 2008).

The utilization of *C. elegans* across genetics, neurology, and medical research is one of the most promising approaches in modern toxicological investigations (Hartman et al., 2021; DDai et al., 2025). The well-characterized stress response pathways of *C. elegans*, including oxidative stress, heat shock, and detoxification mechanisms, provide valuable insights into cellular and molecular responses to xenobiotics. The primary toxic mechanism of organophosphorus pesticides, such as phoxim, involves cholinesterase inhibition; however, there is growing evidence of additional pathways of toxicity, and these remain inadequately characterized. Recent studies have implicated oxidative stress, mitochondrial dysfunction, and endocrine disruption as potential secondary mechanisms underlying organophosphate toxicity (Costa et al., 2008; Slotkin and Seidler, 2009).

The aim of the present study was to comprehensively investigate the effects of phoxim on the model organism *C. elegans*, with an emphasis on three critical physiological processes that may be adversely affected: autophagy, intestinal barrier integrity, and neuronal function. Using transgenic strains and advanced imaging techniques, we sought to elucidate the molecular mechanisms underlying phoxim toxicity beyond cholinesterase inhibition. These findings contribute to a more comprehensive understanding of organophosphate pesticide toxicity and potentially inform the development of safer agricultural practices and improved risk-assessment strategies.

## 2 Materials and methods

### 2.1 Main instruments and reagents

The main laboratory instruments were as follows: a Ts2R inverted fluorescence microscope (Nikon, Japan), a TD-60 low-speed centrifuge (Sichuan Shuke Instrument Co., Ltd.), a TAdvanced PCR instrument (Biometra, Germany), an SE260 electrophoresis instrument (Bio-Rad, USA), a Gel DOC^TM^ XR + gel imaging system (Bio-Rad, USA), a LightCycler 96 real-time fluorescence quantitative PCR instrument (Eppendorf, Germany), an Allegra X low-temperature high-speed centrifuge (Beckman, USA), 40% phoxim (Shandong Essen Chemical Co., Ltd.), an RNA extraction kit (Beijing Tiangen Biochemical Technology Co., Ltd.), a cDNA synthesis kit (Beijing Tiangen Biochemical Technology Co., Ltd.), SYBR Green (Beijing Tiangen Biochemical Technology Co., Ltd.), a cDNA synthesis kit (Beijing Tigermark Ltd.), and SYBR Green (Beijing Polymeric Biotechnology Co., Ltd.); qPCR primers were synthesized by Shanghai Sangong Bioengineering Co., Ltd. The M9 buffer consisted of 5.8 g of disodium hydrogen phosphate heptahydrate, 3.0 g of disodium dihydrogen phosphate, 5.0 g of sodium chloride, and 0.25 g of magnesium sulfate heptahydrate dissolved and then fixed to 1 L. The buffer was sterilized. DMSO was obtained from Beijing Solarbio Biochemical Technology Co., Ltd.

### 2.2 *C. elegans* strains

Three of the four *C. elegans* lines (N2, *lgg-1*::GFP, BZ555, and CL4176) were provided by the Yunnan State Key Laboratory of Biological Resource Conservation and Utilization. They were actually derived from the *C. elegans* Genetic Center (CGC). The fourth line of *C. elegans*, N2, is a wild type. The mutant line *lgg-1*::GFP was genotyped as sqIs11 [*lgg-1*p::mCherry::GFP::*lgg-1* + rol-6] for autophagic vesicles, the BZ555 genotype was genotyped as egIs1 [*dat-1*p::GFP] for dopamine neurons, and the CL4176 genotype was genotyped as dvIs27 [*myo-3*p::A-Beta (1–42)::*let-8513*′UTR + *rol-6* (su1006)] X. After synchronization, L1 *C. elegans* were grown in a constant-temperature incubator at 15 °C to L4 and then transferred to a constant-temperature incubator at 25 °C to induce the production of Aβ to paralyze the *C. elegans*; the rate of paralysis was considered a measure of the neurotoxicity of phoxim.

### 2.3 *C. elegans* culture and synchronization

NGM (NaCl, peptone, CaCl_2_, Mg_2_SO_4_, phosphate buffer, and agar) culture was based on incubation in a constant-temperature chamber at 20 °C, and the *C. elegans* were cultured until spawning. Synchronization was performed by feeding on *E. coli* OP50 in a 20 °C incubator for 48 h. After a large number of eggs appeared on the plate, the *C. elegans* and eggs were rinsed with M9 solution in a 14-mL collection tube and centrifuged at 3,000 rpm for 2 min. The supernatant was discarded, and 2 mL of *C. elegans* lysing solution was added with high-speed shaking for 1 min. When a large number of worms were broken up in the tube, M9 was added and fixed to 14 mL, and centrifuged at 3,000 rpm for 2 min, and the supernatant was discarded. This was repeated thrice until no sodium hypochlorite odor was present. Subsequently, 1 mL of M9 was transferred by pipette to a 6-cm plate to disperse the eggs, and the plate was placed in an incubator at 20 °C for incubation.

### 2.4 Acute toxicity test: calculation of LC_50_

Following Koch’s quantitation method, NGM media of 1, 2.51, 6.31, 15.85, and 39.81 μg/mL of phoxim at 40% concentration were prepared as the mother liquor. After the *C. elegans* were synchronized, the eggs were collected and incubated to the L1 stage, and the L1 *C. elegans* were added to the normal NGM plates to grow to the L4 stage, after which they were transferred to the NGM plates with different concentrations of phoxim for culture. Between 20 and 25 synchronized individuals were placed into each plate, and each group was set up with three parallel samples. Incubation was carried out in an incubator at a constant temperature of 20 °C for 24 h, and the number of dead *C. elegans* was counted through observation under a microscope. The survival experiment consisted of a short-term acute exposure (24 h). The mortality rate of *C. elegans* was calculated, and the experiments were repeated thrice.

### 2.5 Measuring the mortality rate

After determining the appropriate dose through the pretest, NGM media of 0.5, 1, 2.5, and 5 μg/mL of phoxim at 40% concentration were prepared as the mother liquor. Phoxim was first dissolved in DMSO to prepare a stock solution and then diluted with M9 buffer to the desired concentrations. The final DMSO concentration in all treatments, including the control group, was maintained at 0.1%. The control group is referred to as the blank control throughout the manuscript. After the *C. elegans* were synchronized, the eggs were collected and incubated to the L1 stage, and the L1 individuals were added to the normal NGM plates to grow to the L4 stage and transferred to the NGM plates with different concentrations of phoxim for culturing, and 20–25 synchronized *C. elegans* were placed into each plate, and each group was set up with three parallel samples. The incubation was carried out in an incubator at a constant temperature of 20 °C for 24 h, the number of deaths of the *C. elegans* was observed and recorded under a microscope, the *C. elegans* mortality rate was calculated, and the experiments were repeated thrice.

### 2.6 Measurement of body length and width

After synchronization, C. *elegans* eggs were collected and hatched to obtain L1 larvae. A total of 25 L1-stage individuals were transferred to culture plates containing phoxim at concentrations of 0.5, 1, 2.5, and 5 μg/mL. The L1-stage individuals were also added to the standard NGM plates for culture. After incubation at 20 °C for 24 h, *C. elegans* were observed under a microscope in order to determine their body length and width. The control group is referred to as the Blank control throughout the manuscript was set up for all experiments. Each group included three parallel samples, and all experiments were repeated three times.

### 2.7 RNAi interference assay

The single clone of the *eat-2* gene-interfering bacteria was placed into the LB liquid medium containing ampicillin-resistant bacteria and placed into a shaker at 180 rpm at 37 °C for overnight incubation. A 300-µL volume of bacterial solution was added to the NGM medium containing 1 mmol/L IPTG and 100 g/mL AMP; the mixture was then blow-dried on an ultraclean table and allowed to incubate overnight at 25 °C to induce RNA production. Once *C. elegans* digests the bacterium, the small RNAs expressed in the bacterium will enter the cells of *C. elegans* and interfere with a specific gene to reduce the expression level of the gene. The steps in the experiment are as follows: eggs were collected from synchronized *C. elegans* and incubated to the L1 stage, after which they were added to the ordinary NGM plate and grown to the L4 stage and then transferred to 0.5, 1, 2.5, and 5 μg/mL phoxim NGM medium culture at a *C. elegans* density of 20–25 worms in each plate. Three parallel samples were set up and incubated at 20 °C for 24 h. After incubation, the activity of *C. elegans* was observed under a microscope; the frequency of pharyngeal swallowing was recorded, along with the mortality rate of *C. elegans*. The experiment was repeated thrice.

### 2.8 BZ555 *C. elegans’* nerve cord fluorescence

After synchronization of *C. elegans*, the eggs were collected to hatch to the L1 stage, L1 *C. elegans* were added to ordinary NGM plates or NGM plates with a concentration of 1 μg/mL phoxim and cultured in an incubator at 20 °C for 48 h to the L4 stage, and the preparations were observed under an inverted fluorescence microscope to observe the fluorescence intensity of *C. elegans*, which included secreting dopamine neurons and changes to the nerve cord. There were 20 individual *C. elegans* in each group, and the experiment was repeated thrice. Quantitative analysis of the average number of neuraxes in the same field of view was carried out. Confocal fluorescent images were acquired using an Olympus confocal fluorescence microscope. Image processing and quantitative analysis were performed using Fiji (ImageJ 2, version 1.54 p): 1. image preparation, 2. threshold adjustment, 3. parameter settings, 4. start measurement.

### 2.9 CL4176 *C. elegans’* paralysis rate

L1-stage Alzheimer’s disease (AD) model *C. elegans* CL4176 was cultured at 15 °C in an incubator for 48 h to the L4 stage, after which the *C. elegans* were transferred to NGM plates with 0.5 and 1 μg/mL of phoxim and incubated at 25 °C to induce Aβ production. The rate of paralysis of the *C. elegans* was recorded every 4 h. Three parallel samples were set up in each group, and the procedure was repeated thrice.

### 2.10 FD&C blue #1 enteric staining

FD&C Blue #1 was prepared as a 10% solution using M9 mixed with an equal volume of OP50 and then set aside. L4-stage *C. elegans* were transferred to plain NGM plates containing the egg-laying inhibitor pentafluorouracil, 10 mg/mL oligofructose, and 10 mg/mL inulin; NGM plates containing 0.5, 1, and 2.5 μg/mL of phoxim; and NGM plates containing 1 μg/mL of phoxim +10 mg/mL of oligofructose or 1 μg/mL of phoxim +10 mg/mL of inulin. *C. elegans* were incubated at a constant temperature of 20 °C for 144 h for collection. Based on preliminary experiments, the 144-h pretreatment ensured that the experiments were conducted within an appropriate time window within which the *C. elegans* would display this phenotype. The *C. elegans* were resuspended with an appropriate amount of M9, 200 individuals were aspirated into a new 1.5-mL EP tube, and the *C. elegans* were resuspended by adding an appropriate amount of FD&C Blue #1 OP50 mixture with gentle shaking and then stained at 20 °C for 4–5 h. Intestinal staining was observed under an inverted fluorescence microscope, and the intestines were stained with an intact blue color, indicating that the intestines were not damaged and that intestinal function was normal. The blue dye of the food diffused around the intestines, which indicated that the intestines were leaking and that intestinal function was impaired. Three parallel samples were set up for each group, and the experiment was repeated thrice.

### 2.11 *C. elegans* autophagic vesicle

The *lgg-1*::GFP fluorescent strain of *C. elegans* was synchronized and collected after the eggs were incubated to the L1 stage; these individuals were then added to the normal NGM plates or NGM plates with a concentration of 1 μg/mL phoxim and cultured in an incubator at 20 °C for 48 h to the L4 stage. The number of autophagic microsomes in the intestinal cells was counted using a fluorescent inverted microscope, with 20 *C. elegans* per group, and the experiments were repeated thrice.

### 2.12 Real-time fluorescent quantitative PCR (qPCR)

After synchronization, the eggs were collected to hatch L1 *C. elegans*, which were then added to ordinary NGM plates and NGM plates with 1 μg/mL phoxim for incubation. The *C. elegans were* collected after 48 h. The total RNA of *C. elegans* was extracted following the steps of the RNA extraction kit from Bao Bioengineering (Dalian) Co., Ltd., and cDNA was synthesized by reverse transcription according to the steps of the Reverse Transcription Kit from Tiangen Biochemical Technology Co., Ltd. and subjected to real-time fluorescence PCR. *gpd-1* was used as the internal reference gene, and the 2^−ΔΔCT^ method was used to calculate the total RNA of the 1 μg/mL phoxim-stained group compared with the blank control. The primer sequences are shown in Table 1.

**TABLE 1:** Primer sequences for real-time fluorescence quantitative PCR.

Primer name	Base sequence
*gpd-1*F	TCA​AGG​AGG​AGC​CAA​GAA​GG
*gpd-1*R	CAG​TGG​TGC​CAG​ACA​GTT​G
*vps-34*F	AGT​CGC​AGA​GTT​TGA​AGG​C
*vps-34*R	GAC​GAG​CAA​GTT​GAG​AGG​A
*atg-13*F	GGTGCGAATGGTTCAATC
*atg-13*R	TCC​GAC​AGG​TGG​ATA​ACT​TT
*unc-51*F	GAC​TGC​TCA​AGA​GAA​ACG​C
*unc-51*R	GAA​GCG​AAG​ATT​GTG​GTG​T

### 2.13 Data analysis

Each of the above-described experiments were repeated thrice. Graphpad Prism 8.4.0 was used for graphing and data analysis, and t-test was used for two-by-two comparisons, with a statistically significant difference of *p* < 0.05; the more the *sign, the more significant the difference.

## 3 Results

### 3.1 The effect of different concentrations of phoxim on *C. elegans* survival

**FIGURE 1:**
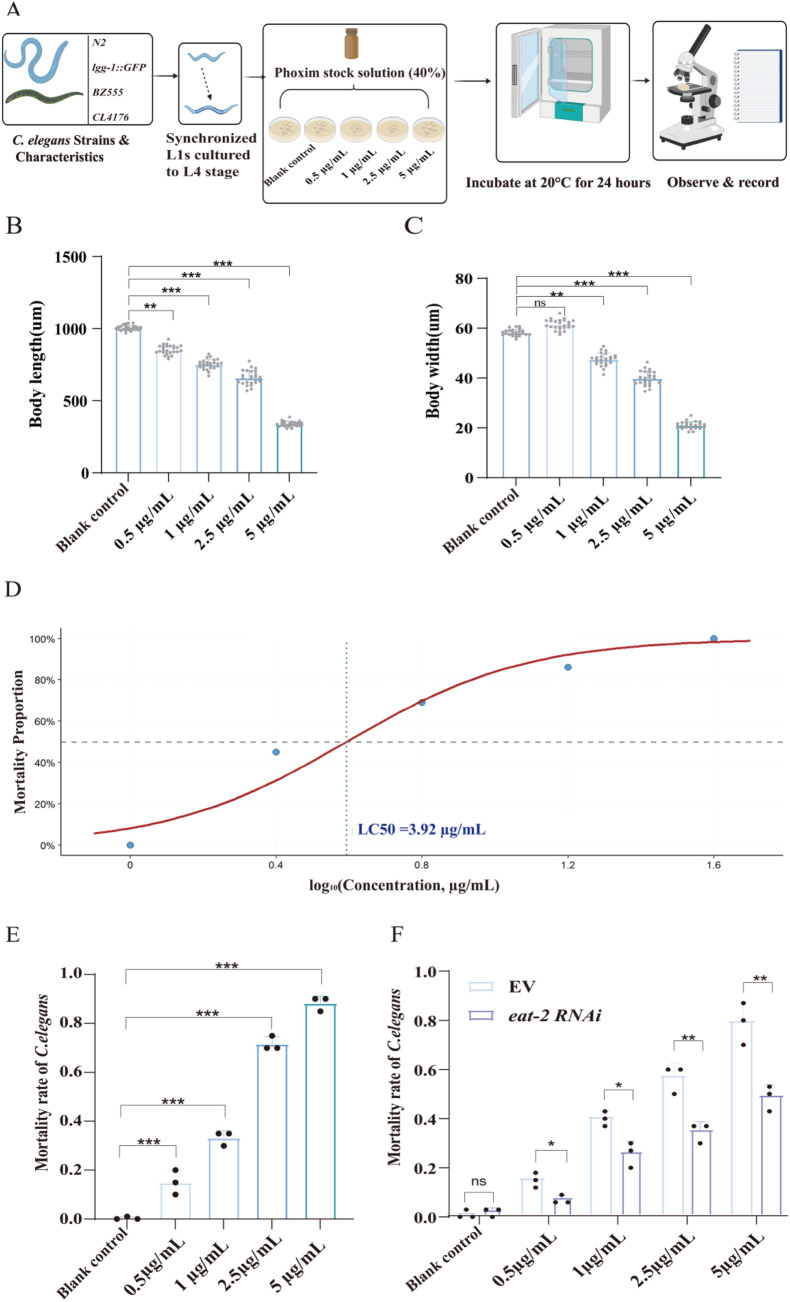
Toxicological effects of phoxim exposure in *C. elegans.* **(A)** Schematic of the experimental design, in which 4 *C. elegans* strains (N2, *lgg-1*::GFP, BZ555, and CL4176) were synchronized, cultured to the L4 stage, and exposed to different concentrations of phoxim for 24 h before phenotypic assessment. **(B,C)** Body length and width of the N2 strain of *C. elegans* decreased significantly with increasing phoxim concentrations. **(D)** Dose–response curve fitted with a logistic regression model, revealing an LC_50_ of 3.92 μg/mL of N2. **(E)** Mortality rates increased markedly with higher phoxim levels. **(F)** *eat-2* mutant showed increased mortality rates as phoxim treatment concentrations increased, but mortality rates were lower than the corresponding values in the EV control. Statistical significance is denoted by *p* < 0.05 (*)*, p < 0.01* (**), and *p* < 0.001 (***), with nonsignificant differences labeled as “ns.” Each dot represents an individual *C. elegans* or biological replicate; error bars indicate standard deviations.

The median concentration of phoxim resulting in 50% mortality of *C. elegans* within 24 h (LC50) was 3.92 μg/mL (Figure 1D). According to existing research, phoxim can affect the behavior and condition of the *C. elegans*. We observed that with increasing concentrations of phoxim, the body length and width of *C. elegans* decreased (*p* < 0.01) (Figures 1B,C). The 24-h mortality rate of *C. elegans* was determined by poisoning with increasing concentrations of phoxim: 0.5, 1, 2.5, and 5 μg/mL. The corresponding mortality rates at 24 h were 17%, 38%, 57%, and 80%, compared with the blank control (containing 0.1% DMSO) (*p* < 0.001) (Figure 1E). To test factors that change the rate of poisoning, the mortality rate of a particular *eat-2* RNAi bacterial strain *C. elegans* was used. This *eat-2* RNAi bacterial strain *C. elegans* is characterized by reduced pharyngeal pumping, resulting in decreased frequency of swallowing in the pharynx. Consequently, mortality was reduced from 17% to 8%, 38% to 22%, 57% to 37%, and 80% to 50% (*p* < 0.05) (Figure 1F). This indicates that the *eat-2* gene inhibited swallowing, leading to a reduction in the uptake of phoxim by the *C. elegans* and, therefore, lower mortality than that of the non-mutant *C. elegans.*

### 3.2 Dose-dependent neurotoxicity of phoxim in *C. elegans*

**FIGURE 2:**
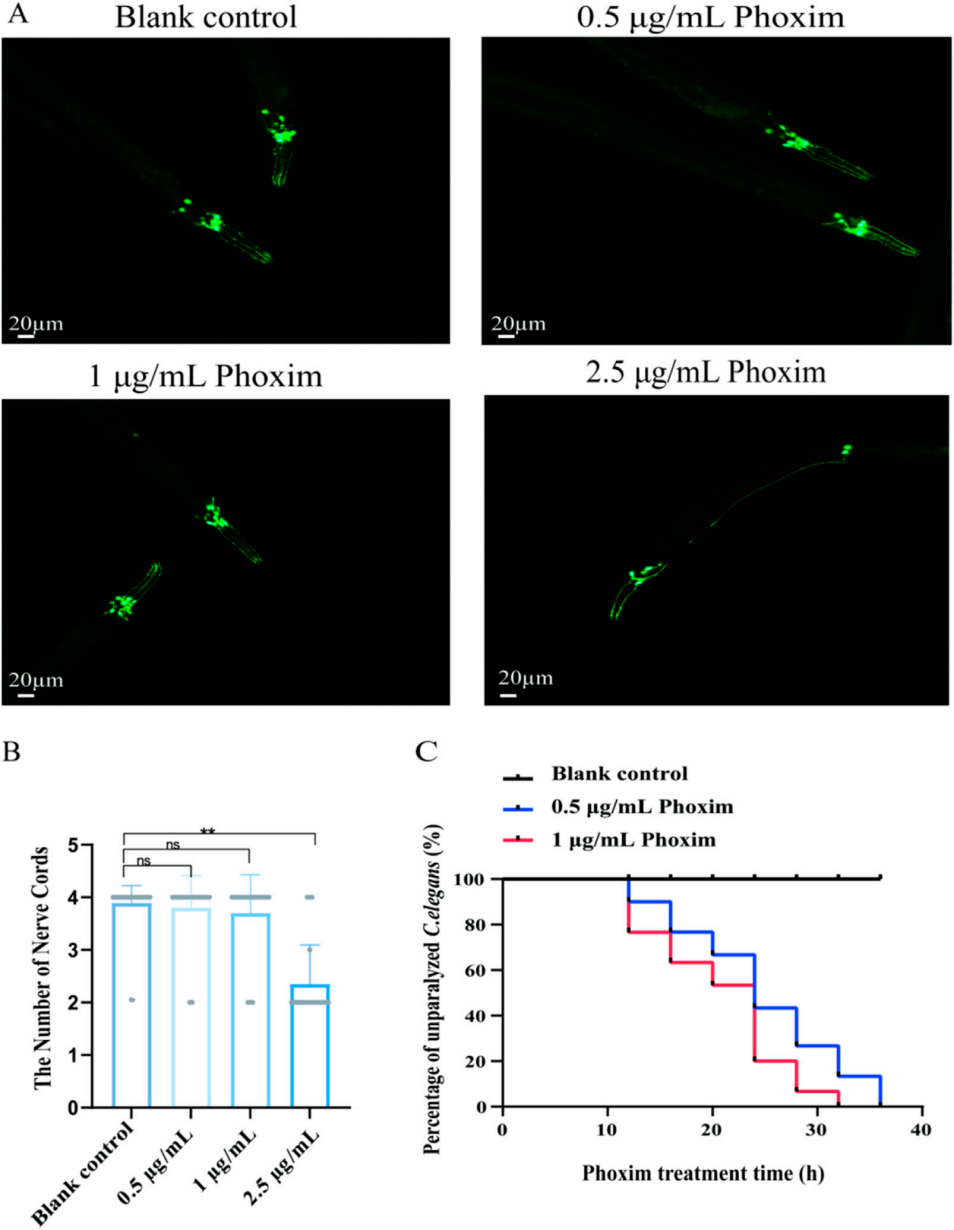
Neurotoxic effects of phoxim on *C. elegans*. **(A)** Fluorescence imaging of BZ555 transgenic *C. elegans* with dopaminergic neuron signals visibly decreasing as phoxim concentration increases, indicating neuronal damage. **(B)** Quantitative analysis showed a significant reduction in fluorescence intensity at 1.0 and 2.5 μg/mL, which is indicative of a dose-dependent neurotoxic effect. **(C)** Results of paralysis assays using CL4176 *C. elegans*; higher phoxim concentrations lead to a faster onset of paralysis, further indicating that phoxim impairs neuromuscular function in a concentration-dependent manner. Statistical significance is denoted by *p* < 0.05 (*), *p < 0.01* (**), and *p* < 0.001 (***), with nonsignificant differences labeled as “ns.” Each dot represents an individual *C. elegans* or biological replicate; error bars indicate standard deviations.

As evidenced by the results of the experiments reported here, phoxim exerts a strong lethal effect on *C. elegans* and exerts a high degree of neurotoxicity. The BZ555 strain of *C. elegans* with the mutant gene *dat-1*:GFP was used to observe the changes in dopamine neurons. The CL4176 strain, a model of AD, was induced at 25 °C, which resulted in Aβ production, causing paralysis of the *C. elegans*. The effects of phoxim staining on the dopamine-secreting neurons and AD symptoms were assessed using the BZ555 and CL4176 strains, respectively. Neural cord changes in dopamine-secreting neurons were observed in the BZ555 strain treated with different concentrations of phoxim. Compared with the blank control, *C. elegans* treated with 0.5 and 1 μg/mL phoxim showed no significant changes in the number of nerve cords, whereas *C. elegans* treated with 2.5 μg/mL phoxim showed a reduction in the number of dopamine-secreting neurons, and the change was from 4 to 2 (*p* < 0.01) (Figures 2A,B). In addition, the effect of different concentrations of phoxim on the rate of paralysis in the AD model *C. elegans* (strain CL4176) was examined: paralysis occurred in all CL4176 *C. elegans* that had ingested phoxim at 0.5 and 1 μg/mL (100% of *C. elegans* paralyzed at 32 and 36 h) compared to no paralysis within 40 h in the blank control (*p* < 0.05), suggesting that Aβ-induced paralysis was significantly accelerated by the ingestion of phoxim (Figure 2C). Based on our results, we suggest that phoxim-poisoning damaged the dopamine-secreting neurons and accelerated Aβ-induced paralysis.

### 3.3 Phoxim is associated with autophagy inhibition, which may contribute to *C. elegans* neurotoxicity mechanism

**FIGURE 3:**
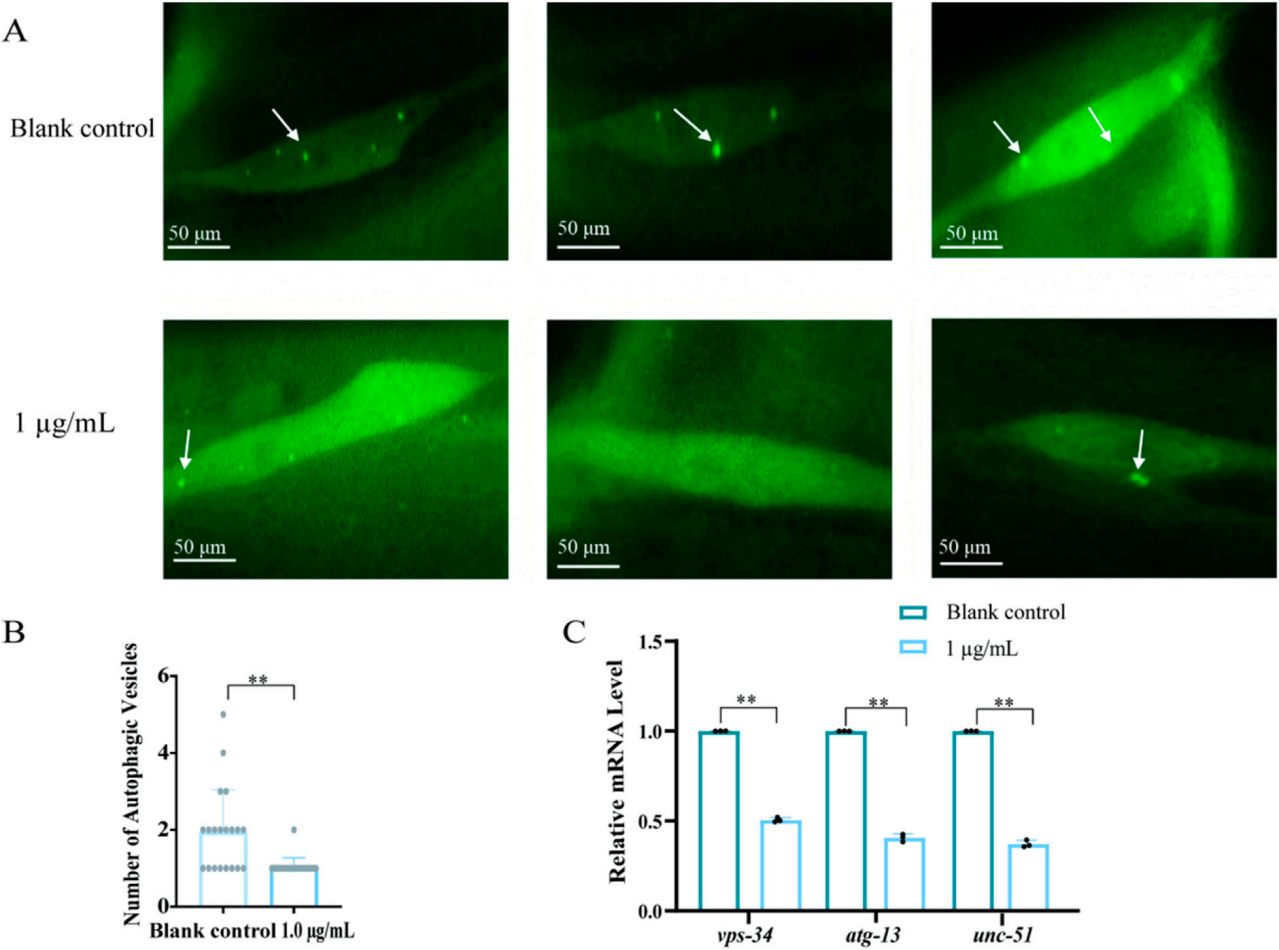
Phoxim inhibits autophagy in *C. elegans*. **(A)** *lgg-1*::GFP *C. elegans* treated with phoxim showed autophagosomes in intestinal cells; white arrows indicate autophagosomes. **(B)** Changes in the number of autophagosomes in intestinal cells of *C. elegans* treated with phoxim. **(C)** Changes in the expression of *vsp-34*, *atg-13*, and *unc-51* in *C. elegans* treated with 1 μg/mL phoxim. Statistical significance is denoted by *p* < 0.05 (*), *p < 0.01* (**), and *p* < 0.001 (***), with nonsignificant differences labeled as “ns.” Each dot represents an individual *C. elegans* or biological replicate; error bars indicate standard deviations.

*Lgg-1*::GFP is a transgenic strain of *C. elegans w*ith the LGG-1 protein fused to green fluorescent protein (GFP), enabling direct visualization of autophagy via fluorescence microscopy. Autophagy can be quantified by counting GFP puncta, making this strain a standard tool for autophagy research. Unlike the wild-type *C. elegans*, which has an unmodified LGG-1 protein that cannot be visualized by fluorescence, the *lgg-1*::GFP variant allows research workers to directly observe and measure autophagy dynamics during development, aging, and disease processes (Chen et al., 2017). The pathogenic bacterium PA14 infested *C. elegans* and inhibited autophagy, leading to increased mortality from intestinal damage, as well as intestinal damage related to autophagy caused by the ingestion of phoxim. Observing the changes of autophagic vesicles detected in *lgg-1*::GFP *C. elegans*, compared with the blank control, the average number of autophagic vesicles in *C. elegans* intestinal cells was 1.8, and the average number of autophagic vesicles in the intestinal cells was 1.3 in phoxim-infected *C. elegans* at 1 μg/mL, which was a significant decrease in the number of intestinal cellular autophagic vesicles. The decrease in the number of autophagic vesicles was particularly significant in some *C. elegans* individuals, and cells that did not form autophagosomes were present (*p* < 0.05) (Figures 3A,B). In order to verify the inhibition of autophagy by phoxim, the transcript levels of autophagy-associated genes, namely, *vps-34*, *atg-13*, and *unc-51*, were detected in *C. elegans* treated with 1 μg/mL of phoxim. qPCR assay revealed that compared with the blank control, the level of expression of *vsp-34*, *atg-13*, and *unc-51* was downregulated by 0.53-, 0.43-, and 0.36-fold, respectively (*p* < 0.05) (Figure 3C). Based on our results, the inhibition of *vps-34*, *atg-13*, and *unc-51* expression by phoxim led to a reduction in the autophagy levels in intestinal cells.

### 3.4 Intestinal damage induced by phoxim

**FIGURE 4:**
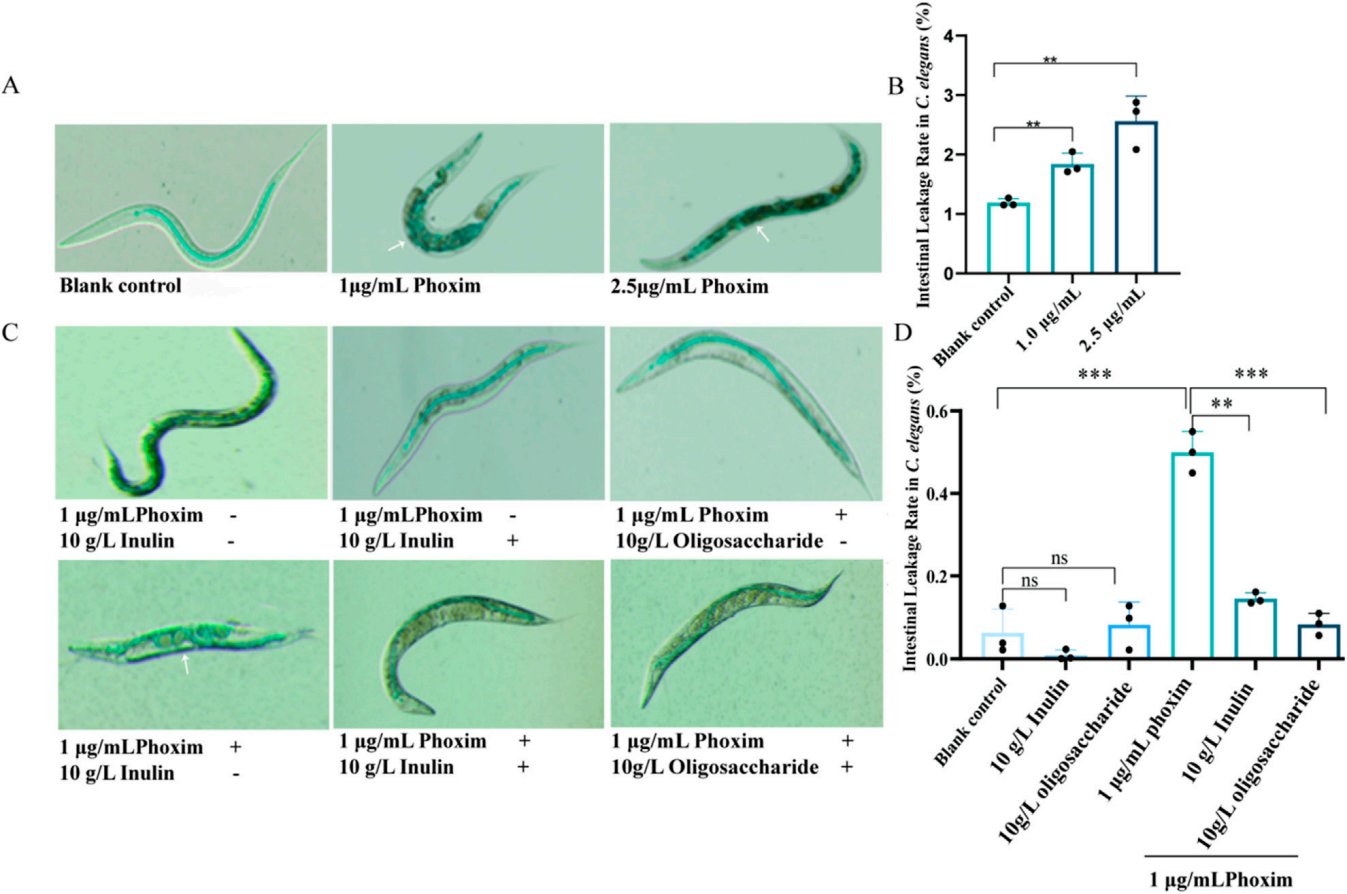
Effect of phoxim on the intestinal damage of *C. elegans*. **(A)** Intestinal staining with FD&C Blue #1 dye in *C. elegans* treated with different concentrations of phoxim. White arrows indicate intestinal damage. **(B)** Percentage of intestinal leakage in worms treated with different concentrations of phoxim. **(C)** Intestinal staining with FD&C Blue #1 dye in *C. elegans* treated with different concentrations of phoxim and supplemented with soluble dietary fiber (fructo-oligosaccharides and inulin), where “-” represents “untreated” and “+” represents “treated.” **(D)** Percentage of intestinal leakage in worms treated with different concentrations of phoxim and supplemented with soluble dietary fiber (fructo-oligosaccharides and inulin). Statistical significance is denoted by *p* < 0.05 (*), *p < 0.01* (**), and *p* < 0.001 (***), with nonsignificant differences labeled as “ns.” Each dot represents an individual *C. elegans* biological replicate; error bars indicate standard deviations.

The intestine of *C. elegans* is closely related to immune regulation, substance metabolism, the occurrence of many diseases, and the aging process of the organism; therefore, it is especially important to detect intestinal damage induced by phoxim on *C. elegans*. The effects of different concentrations of phoxim on the *C. elegans* intestines were detected by FD&C Blue #1 staining, and the mature *C. elegans* were stained in the intestine after being reared in media containing different phoxim concentrations for 144 h. The results showed that the intestines were damaged to different degrees by phoxim at different concentrations. Compared with 5% of *C. elegans* showing intestinal damage with the blank control, 46.7% and 68.3% of *C. elegans* intestines were damaged by phoxim staining at 1 and 2.5 μg/mL, respectively (*p* < 0.05) (Figures 4A,B).

Soluble dietary fiber supplements can modulate the intestinal flora diversity, protect intestinal integrity, and reduce inflammation. In order to test whether soluble dietary fiber has a protective effect on the intestinal tract during phoxim contamination, 10 g/L oligofructose or inulin (soluble dietary fiber) was added to the contamination medium, and the proportion of *C. elegans* with intestinal damage decreased to 12% and 15% with the 10 g/L oligofructose or inulin treatment, respectively, compared with 50% of *C. elegans* intestinal damage with 1 μg/mL (*p < 0.05*) (Figures 4C,D). Thus, dietary fiber had a protective effect against intestinal damage caused by phoxim. In conclusion, long-term intake of either high or low doses of phoxim resulted in intestinal leakage and damage in *C. elegans*, and soluble dietary fiber had a protective effect against phoxim-mediated intestinal damage.

## 4 Discussion

*C*. *elegans* serves as an ideal biological model that possesses unique advantages in genetic, neuroscience, and toxicological research. Since the 1990s, research workers have recognized the immense potential of this species as a research tool in environmental toxicological assessment Grandjean and Landrigan (2014) identified a comprehensive list of developmental neurotoxicants that are evident in *C. elegans*, including heavy metals, organic compounds, pesticides, and other substances. The toxicity of these substances manifests through behavioral and neurological impairments. Specifically, organophosphate pesticides function as neurotoxic agents capable of progressively weakening and ultimately eliminating normal biological behavioral functions, such as locomotion and feeding behaviors (Kim and Kang, 2021). Given that it is essential for survival, feeding serves as a primary behavioral assessment indicator in toxicological studies of *C. elegans*. The feeding organ of *C. elegans* is the pharynx, which is a simple neuromuscular pump that completes feeding via muscle contraction and relaxation. With the emergence of feeding indicators, research utilizing foraging and feeding behavior indicators for neurotoxic agents has gradually developed (Ishita et al., 2020; Sherman and Harel, 2024; Cao et al., 2023). In this study, we utilized a strain of *C. elegans* containing the *eat-2* mutant, a gene encoding acetylcholine receptor subunits that regulate neuronal function, thereby weakening the swallowing capability of the affected *C. elegans*. The results showed that *eat-2* RNAi-treated *C. elegans* exhibited significantly reduced mortality under phoxim exposure, indicating that oral ingestion is the primary route of phoxim absorption, which reduces phoxim intake and consequently increases the worm survival rates. However, it is crucial to note that the *eat-2* gene not only affects feeding processes but also participates in neuromuscular function regulation, potentially causing interactive interference in paralysis and survival assessments (Gao et al., 2018). Specifically, the *eat-2* gene regulates worm sensitivity to organophosphate pesticide neurotoxicity. Reduced *eat-2* function might indirectly influence worm toxicant sensitivity by altering neural signal transmission rather than solely protecting it through reduced intake (Wang, 2019). Therefore, future studies should consider using more specific feeding control genes or developing more precise quantitative feeding measurement methods to better distinguish between reduced intake and neuroprotective effects.

Impaired growth and reproduction are common manifestations of neurotoxicity (Wang et al., 2023). Variations in body length can effectively reflect the developmental rate and physiological status of *C*. *elegans* (Stojanovski et al., 2022). Here, we report that as phoxim concentration increased, it progressively inhibited the growth of *C. elegans*. The body width and length ratio of the treated group became significantly shorter than those of the blank control, indicating that phoxim exhibits a certain level of neurotoxicity in *C. elegans*. Thus, we systematically evaluated the impact of monocrotophos on the nervous system of *C. elegans* using two transgenic models, BZ555 and CL4176. The BZ555 variant carries the *dat-1*:GFP reporter gene, which enables the labeling of dopaminergic neurons. Dopaminergic neurons are critical for maintaining normal motor capabilities and behavioral coordination, and their damage may explain the reduced motor function following exposure to monocrotophos. CL4176 is an AD *C. elegans* model in which temperature induction leads to Aβ production in muscle cells, causing *C. elegans* paralysis. Based on our experimental results, we believe that this finding is significant and suggests that exposure to environmental organophosphate pesticides is a potential risk factor for neurodegenerative diseases.

Autophagy is a highly conserved intracellular metabolic process involving the formation of double-membrane autophagosomes that engulf cytoplasmic components and transfer them to lysosomes for degradation (Chen et al., 2023; Liu et al., 2023; Mochida and Nakatogawa, 2022). In *C. elegans*, autophagy involves four enzyme complexes: a serine/threonine protein kinase complex (UNC-51 and ATG-13) inducing autophagy activity, a class-III phosphatidylinositol 3-kinase complex (BEC-1 and VPS-34) initiating vesicle formation, and two ubiquitin-like conjugation pathways (ATG-3, ATG-4, ATG-7, and LGG-1) promoting autophagosome expansion and completion. The intestine serves as the primary metabolic tissue in *C. elegans* and is a critical regulator of systemic environmental stability and lifespan (Baxi and de Carvalho, 2018). Our research found that upon phoxim exposure, significant intestinal barrier damage occurred, which was manifested by blue dye leakage into surrounding tissues. Molecular mechanism investigations revealed that phoxim exposure significantly reduced autophagy-related gene expression (*vps-34*, *atg-13*, and *unc-51*) to 0.53, 0.43, and 0.36 times of the control levels, respectively. Simultaneously, the autophagic vesicle count in *lgg-1*::GFP transgenic worms decreased from 1.8 to 1.3. These results suggest that phoxim induces intestinal cell dysfunction by suppressing autophagy pathways, thereby disrupting the intestinal barrier integrity. Notably, in studies of pathogen infection of *C. elegans*, inhibiting autophagy does not affect the accumulation of *Pseudomonas aeruginosa* in the intestine but instead induces intestinal necrosis (Zou et al., 2014). This finding echoes our observations, indicating that autophagy plays a crucial role in maintaining intestinal cell health and defending against harmful exogenous substances. Of course, the autophagy process assessment in this study was conducted only at a single concentration (1 μg/mL), without establishing a dose–effect relationship, making it difficult to comprehensively elucidate the dose-dependent effects of fenitrothion on autophagy pathways. The lack of time-series observations of dynamic changes in autophagy indicates that single time-point measurements may miss critical biological processes. No mechanism verification experiments for autophagy pathways were performed, such as using autophagy agonists/inhibitors for intervention. Thus, we were unable to confirm whether the observed changes were directly regulated by the autophagy pathway. This provided a foundation for subsequent in-depth investigations.

Based on the discovery of FNT-induced intestinal damage, we explored potential protective strategies. Soluble dietary fibers have been proven to have the ability to regulate intestinal microbiota diversity, protect intestinal integrity, and reduce intestinal inflammation, and are widely present in various natural plants, such as Jerusalem artichoke, wheat, barley, rye, onion, garlic, asparagus, and banana (Wang et al., 2020; Bornet and Brouns, 2002). Our research found that adding soluble dietary fibers (such as fructo-oligosaccharides or inulin) to fenitrothion-containing *C. elegans* culture medium effectively mitigated fenitrothion-induced intestinal damage. This protective effect may be achieved through multiple mechanisms. First, studies have shown that soluble dietary fibers such as short-chain fructo-oligosaccharides and inulin can significantly increase the relative abundance of *Bifidobacterium* and reduce the proportion of potential pathogenic bacteria in the intestine (Holscher et al., 2015). This change in microbial composition may alleviate fenitrothion toxicity through the following mechanisms: beneficial bacteria may participate in the biodegradation or transformation of fenitrothion (Trinder et al., 2016), and increased microbial diversity enhances intestinal ecosystem stability and resistance to exogenous toxins (Flint et al., 2017). Second, dietary fiber might reverse fenitrothion-induced inhibition of autophagy pathways by enhancing autophagy activity. Finally, short-chain fatty acids produced by dietary fibers may directly strengthen the intestinal barrier function. Studies have demonstrated that soluble dietary fibers, such as inulin, significantly improve intestinal barrier function disorders caused by various chemicals by increasing the production of SCFAs (Liu et al., 2015). From an integrated mechanism perspective, the protective effects of dietary fiber may be closely related to the restoration of autophagic function. Previous research has shown that inulin activates autophagy through the AMPK/mTOR signaling pathway, thereby alleviating intestinal damage caused by a high-fat diet (Li et al., 2023). In the *C. elegans* model, fructo-oligosaccharides were confirmed to upregulate the expression of the key autophagy gene *lgg-1*, increase autophagy flux, extend worm lifespan, and enhance resistance to environmental stress (Zhang et al., 2019). Considering our findings that fenitrothion significantly suppressed the expression of autophagy-related genes (*vps-34*, *atg-13*, and *unc-51*) and reduced the number of autophagosomes, soluble dietary fiber may exert protective effects by reversing this suppression.

The findings reported here reveal the mechanisms of the toxic effects of phoxim on *C*. *elegans* through multi-system assessment, including neurological damage (dopaminergic neuron degeneration and enhanced Aβ-related toxicity) and intestinal barrier dysfunction (through the inhibition of autophagy pathways). These findings expand our understanding of the mechanisms of organophosphate toxicity. Moving forward, we plan to (1) further clarify the dual role of the *eat-2* gene in phoxim toxicity (reduced feeding and regulation of neuromuscular function); (2) explore in depth the molecular mechanisms of the protective effects of dietary fiber, especially its interaction with autophagy pathways; and (3) conduct translational research to evaluate the potential impacts of long-term, low-dose phoxim exposure on the mammalian nervous system and intestinal health, providing scientific evidence for the safe use of pesticides.

## Data Availability

The original contributions presented in the study are included in the article/Supplementary Material; further inquiries can be directed to the corresponding authors.

## References

[B1] Baxi K. de Carvalho C. E. (2018). Assessing lysosomal alkalinization in the intestine of live Caenorhabditis elegans. J. Visualized Experiments JoVE (134), 57414. 10.3791/57414 29708551 PMC5933508

[B2] Bornet F. R. Brouns F. (2002). Immune-stimulating and gut health-promoting properties of short-chain fructo-oligosaccharides. Nutr. Reviews 60 (10 Pt 1), 326–334. 10.1301/002966402320583442 12392149

[B3] Cao X. Xie Y. Yang H. Sun P. Xue B. Garcia L. R. (2023). EAT-2 attenuates *C. elegans* development via metabolic remodeling in a chemically defined food environment. Cell. Mol. Life Sci. 80 (8), 205. 10.1007/s00018-023-04849-x 37450052 PMC11072272

[B4] Chen Y. Scarcelli V. Legouis R. (2017). Approaches for studying autophagy in Caenorhabditis elegans. Cells 6 (3), 27. 10.3390/cells6030027 28867808 PMC5617973

[B5] Chen T. Tu S. Ding L. Jin M. Chen H. Zhou H. (2023). The role of autophagy in viral infections. J. Biomedical Science 30 (1), 5. 10.1186/s12929-023-00899-2 36653801 PMC9846652

[B47] Costa L. G. Giordano G. Guizzetti M. Vitalone A. (2008). Neurotoxicity of pesticides: a brief review. Front Biosci. 13, 1240–1249. 10.2741/2758 17981626

[B6] Ddai L. Bao H. Yu L. (2025). Antioxidant defense response mediated by DAF-16 attenuates toxicity of herbicides glyphosate and glufosinate ammonium on Caenorhabditis elegans. Biochem. Biophysical Research Communications 756, 151577. 10.1016/j.bbrc.2025.151577 40056504

[B7] Flint H. J. Duncan S. H. Louis P. (2017). The impact of nutrition on intestinal bacterial communities. Curr. Opinion Microbiology 38, 59–65. 10.1016/j.mib.2017.04.005 28486162

[B8] Gao P. Huang G. F. Xie X. L. Huang H. Liu W. X. Yang J. L. (2014). Research progress on analytical methods for organophosphorus pesticide residues in aquatic products. Guangdong Agric. Sci. 41 (15), 83–88. 10.16768/j.issn.1004-874x.2014.15.009

[B9] Gao A. W. Smith R. L. van Weeghel M. Kamble R. Janssens G. E. Houtkooper R. H. (2018). Identification of key pathways and metabolic fingerprints of longevity in *C. elegans* . Exp. Gerontology 113, 128–140. 10.1016/j.exger.2018.10.003 30300667 PMC6224709

[B10] Grandjean P. Landrigan P. J. (2014). Neurobehavioural effects of developmental toxicity. Lancet. Neurology 13 (3), 330–338. 10.1016/S1474-4422(13)70278-3 24556010 PMC4418502

[B11] Guan F. L. Xiong L. F. Fang H. S. Sun Y. T. Jiang X. J Liu W., M. (2020). Acute toxicity of phoxim and mebendazole to Paramisgurnus dabryanus. Ecol. Sci. 39 (06), 25–29.

[B12] Hartman J. H. Widmayer S. J. Bergemann C. M. King D. E. Morton K. S. Romersi R. F. (2021). Xenobiotic metabolism and transport in *Caenorhabditis elegans* . J. Toxicology Environmental Health. Part B, Crit. Reviews 24 (2), 51–94. 10.1080/10937404.2021.1884921 33616007 PMC7958427

[B49] He X. L. Nie Y. Wang N. Xia H. N. Liu J. Y. Lu Y. X. (2012). Current status of organophosphorus pesticide pollution and prevention and control strategies. Environmental Ecology 3 (10), 38–43.

[B13] Holscher H. D. Bauer L. L. Gourineni V. Pelkman C. L. Fahey G. C. Jr Swanson K. S. (2015). Agave inulin supplementation affects the fecal microbiota of healthy adults participating in a randomized, double-blind, Placebo-controlled, crossover trial. J. Nutrition 145 (9), 2025–2032. 10.3945/jn.115.217331 26203099

[B14] Ishita Y. Chihara T. Okumura M. (2020). Serotonergic modulation of feeding behavior in Caenorhabditis elegans and other related nematodes. Neurosci. Research 154, 9–19. 10.1016/j.neures.2019.04.006 31028772

[B45] Kaletta T. Hengartner M. O. (2006). Finding function in novel targets: C. elegans as a model organism. Nat. Rev. Drug Discov. 5 (5), 387–398. 10.1038/nrd2031 16672925

[B15] Kim H. M. Kang J. S. (2021). Metabolomic studies for the evaluation of toxicity induced by environmental toxicants on model organisms. Metabolites 11 (8), 485. 10.3390/metabo11080485 34436425 PMC8402193

[B46] Leung M. C. Williams P. L. Benedetto A. Au C. Helmcke K. J. Aschner M. (2008). Caenorhabditis elegans: an emerging model in biomedical and environmental toxicology. Toxcol. Sci. 106 (1), 5–28. 10.1093/toxsci/kfn121 18566021 PMC2563142

[B118] Li H. Li D. Li L. J. Wu N. (2023). Study on phoxim-induced metabolic changes in crucian carp based on ∼1H-NMR metabolomics. Food & Machinery 39 (05), 21–26. 10.13652/j.spjx.1003.5788.2023.60010

[B18] Li S. Liu X. Y. (2019). Effects of phoxim on CYP3A enzyme activity, mRNA and protein expression in crucian carp liver microsomes. Freshw. Fish. 49 (04), 102–107. 10.13721/j.cnki.dsyy.2019.04.015

[B22] Lin B. X. Yu Y. Wang W. J. (2011). Alcoholysis kinetics and mechanism of organophosphorus pesticide phoxim. J. Agro-Environment Sci. 30 (01), 198–204.

[B23] Liu T. W. Park Y. M. Holscher H. D. Padilla J. Scroggins R. J. Welly R. (2015). Physical activity differentially affects the cecal microbiota of ovariectomized female rats selectively bred for high and low aerobic capacity. PloS One 10 (8), e0136150. 10.1371/journal.pone.0136150 26301712 PMC4547806

[B24] Liu J. Liu Y. Wang Y. Li C. Xie Y. Klionsky D. J. (2023). TMEM164is a new determinant of autophagy-dependent ferroptosis. Autophagy 19 (3), 945–956. 10.1080/15548627.2022.2111635 35947500 PMC9980451

[B26] Mochida K. Nakatogawa H. (2022). ER-phagy: selective autophagy of the endoplasmic reticulum. EMBO Reports 23 (8), e55192. 10.15252/embr.202255192 35758175 PMC9346472

[B27] Okoro N. O. Odiba A. S. Osadebe P. O. Omeje E. O. Liao G. Fang W. (2021). Bioactive phytochemicals with anti-aging and lifespan extending potentials in *Caenorhabditis elegans* . Mol. Basel, Switz. 26 (23), 7323. 10.3390/molecules26237323 34885907 PMC8658929

[B48] Slotkin T. A. Seidler F. J. Wu C. MacKillop E. A. Linden K. G. (2009). Ultraviolet photolysis of chlorpyrifos: developmental neurotoxicity modeled in PC12 cells. Environ. Health Perspect. 117 (3), 338–343. 10.1289/ehp.11592 19337505 PMC2661900

[B28] Shang B. D. Zhang X. P. (2015). Acute toxicity of five insecticides to goldfish. Guizhou Agric. Sci. 43 (09), 138–141.

[B29] Sherman D. Harel D. (2024). Deciphering the underlying mechanisms of the pharyngeal pumping motions in Caenorhabditis elegans. Proc. Natl. Acad. Sci. U.S.A. 121 (7), e2302660121. 10.1073/pnas.2302660121 38315866 PMC10873627

[B30] Stojanovski K. Großhans H. Towbin B. D. (2022). Coupling of growth rate and developmental tempo reduces body size heterogeneity in *C. elegans* . Nat. Commun. 13 (1), 3132. 10.1038/s41467-022-29720-8 35668054 PMC9170734

[B31] Tang S. Z. Chen Z. X. Bai S. Y. Wang H. T. Hao Q. R. Wu S. (2015). Acute toxicity of five aquaculture drugs to juvenile rainbow trout. Chin. J. Fish. 28 (06), 33–37.

[B33] Trinder M. McDowell T. W. Daisley B. A. Ali S. N. Leong H. S. Sumarah M. W. (2016). Probiotic Lactobacillus rhamnosus reduces organophosphate pesticide absorption and toxicity to Drosophila melanogaster. Appl. Environmental Microbiology 82 (20), 6204–6213. 10.1128/AEM.01510-16 27520820 PMC5068162

[B34] Wang D. (2019). “Intestinal barrier for C.elegans against toxicity of environmental toxicants or stresses,” in Target organ toxicology in Caenorhabditis elegans (Singapore: Springer Singapore), 71–95.

[B35] Wang H. He C. Liu Y. Zhao H. Long L. Gai X. (2020). Soluble dietary fiber protects intestinal mucosal barrier by improving intestinal flora in a murine model of sepsis. Biomed. & pharmacotherapy = Biomedecine & Pharmacotherapie 129, 110343. 10.1016/j.biopha.2020.110343 32593968

[B36] Wang Y. Gai T. Zhang L. Chen L. Wang S. Ye T. (2023). Neurotoxicity of bisphenol A exposure on Caenorhabditis elegans induced by disturbance of neurotransmitter and oxidative damage. Ecotoxicol. Environmental Safety 252, 114617. 10.1016/j.ecoenv.2023.114617 36758510

[B37] Xu X. D. Cao Y. H. Deng Y. H. Zhu E. Q. Zeng Q. X. Zhang J. M. (2013). Acute toxicity Test of six common fishery drugs on juvenile Ancherythroculter nigrocauda. Fish. Sci. 32 (12), 696–700. 10.16378/j.cnki.1003-1111.2013.12.001

[B38] Yu F. R. Li D. L. (2015). Impact of organophosphorus pesticides on human health and research progress on pesticide residue detection methods. Ecol. Sci. 34 (03), 197–203. 10.14108/j.cnki.1008-8873.2015.03.031

[B39] Yu X. Li H. Lin D. Guo W. Xu Z. Wang L. (2021). Ginsenoside prolongs the lifespan of *C. elegans* via lipid metabolism and activating the stress response signaling pathway. Int. Journal Molecular Sciences 22 (18), 9668. 10.3390/ijms22189668 34575832 PMC8465798

[B41] Zhang X. C. Zhu X. P. Liu Y. H. Hong X. Y. Huang J. C. Zeng M. L. (2015). Acute toxicity of mebendazole, deltamethrin and copper sulfate to American shad. South China Fish. Sci. 11 (02), 66–71.

[B42] Zhang J. Lian B. Shang W. Li C. Meng X. (2019). Transcriptomic analyses of the anti-aging effect of dietary supplementation with fructooligosaccharides in Caenorhabditis elegans. Food & Funct. 10 (11), 7299–7310.

[B43] Zhang J. Sun Y. Song W. Shan A. (2023). Vitamin E-Inhibited Phoxim-Induced renal oxidative stress and mitochondrial apoptosis *in vivo* and *in vitro* of piglets. Antioxidants Basel, Switz. 12 (11), 2000. 10.3390/antiox12112000 38001853 PMC10668979

[B44] Zou C. G. Ma Y. C. Dai L. L. Zhang K. Q. (2014). Autophagy protects *C. elegans* against necrosis during Pseudomonas aeruginosa infection. Proc. Natl. Acad. Sci. U. S. A. 111 (34), 12480–12485. 10.1073/pnas.1405032111 25114220 PMC4151725

